# Widerstände gegen Präventionsmaßnahmen während der COVID-19-Pandemie: Ursachen und Strategien für ihre Minimierung

**DOI:** 10.1007/s11553-022-00960-2

**Published:** 2022-06-20

**Authors:** Matthias R. Hastall, Viviane Scherenberg

**Affiliations:** 1 Fakultät Rehabilitationswissenschaften, Fachgebiet Qualitative Forschungsmethoden und strategische Kommunikation für Gesundheit, Inklusion und Teilhabe, Technische Universität Dortmund, Emil-Figge-Str. 50, 44227 Dortmund, Deutschland; 2 Fachbereich Public Health und Umweltgesundheit, APOLLON Hochschule der Gesundheitswirtschaft GmbH, Universitätsallee 18, 28359 Bremen, Deutschland

**Keywords:** Compliance-Verhalten, Psychologische Reaktanz, Präventionsverhalten, Gesundheitskommunikation, Corona, Compliance behavior, Psychological reactance, Prevention behavior, Health communication, Corona

## Abstract

**Hintergrund:**

Während der COVID-19-Pandemie („coronavirus disease 2019“) kam es immer wieder zu Widerständen gegenüber nachweislich wirksamen Präventionsmaßnahmen. Eine solche durch Verärgerung und negativen Kognitionen gekennzeichnete „Reaktanz“ erleben Menschen (gemäß der psychologischen Reaktanztheorie) bei einer wahrgenommenen Bedrohung subjektiv wichtiger Freiheiten oder wahrgenommenen Versuchen, ihre Einstellungen oder ihr Verhalten zu ändern.

**Fragestellung:**

Der vorliegende Beitrag beleuchtet die Rolle defensiver Prozesse im Kontext der COVID-19-Pandemie aus der Perspektive einer evidenzbasierten und abwehrsensiblen Risiko- und Krisenkommunikation. Nach einem Überblick über wesentliche Auslöser und Ausprägungen werden Möglichkeiten zur Minimierung von Abwehr diskutiert.

**Ergebnis:**

Widerstände sind in einem gewissen Umfang immer zu erwarten, lassen sich aber durch bestimmte formale und inhaltliche Gestaltungen der Informationen minimieren. Hierzu zählen beispielsweise eine professionelle Anmutung, eine respektvoll wertschätzende und stigmasensible Grundhaltung, eine positive und selbstwirksamkeitsstärkende Ansprache sowie eine Vermeidung emotional überfordernder Informationen wie z. B. stark negative emotionale Appelle oder starkes Verlust-Framing.

**Schlussfolgerung:**

Akteure sollten müssen sich darüber im Klaren darüber sein, dass Abwehrmechanismen durch die Kommunikation sowohl gefördert als auch reduziert werden können. Sie sollten wesentliche Auslöser hierfür kennen und durch eine konsistente, verständliche und adressatengerechte Kommunikation dazu beitragen, Unsicherheiten, Widerstände und Irritationen zu vermeiden.

## Abstract (en)

**Background:**

During the coronavirus disease 2019 (COVID-19) pandemic, resistance against demonstrably effective preventive measures were frequently observed. Such “reactance,” characterized by anger and negative cognitions, is experienced (according to psychological reactance theory) when a threat to subjectively important freedoms or attempts to change attitudes or behaviors is perceived.

**Objectives:**

This paper discusses the role of defensive processes in the context of the COVID-19 pandemic from the perspective of an evidence-based and defense-sensitive risk and crisis communication. Triggers and influencing factors of defensive processes are discussed, as well as techniques for minimizing these unwanted reactions.

**Results:**

Defensive reactions should always be anticipated, at least to some extent, but can be reduced through a defense-sensitive communication design. This includes a professional presentation, a respectful and stigma-sensitive approach, positive and efficacy-bolstering information, and by avoiding emotionally overwhelming information such as strong negative emotional appeals or strong loss framings.

**Conclusion:**

Communicators should be aware that defensive reactions can be triggered as well as minimized through certain features of risk communication. A consistent, clear, and tailored communication approach is needed to reduce uncertainties, defensiveness, and irritation.

Widerstände gegenüber Verhaltensempfehlungen sind ein natürliches und weit verbreitetes Phänomen, gleichzeitig aber eine große Barriere für Präventionsbemühungen. Daher ist es wichtig, die Ursachen und Ausprägungen derartiger Widerstände sowie Möglichkeiten ihrer Minimierung zu verstehen. Der vorliegende Beitrag gibt einen Einblick in das psychologische Phänomen und liefert am Beispiel der COVID-19-Pandemie („coronavirus disease 2019“) Erklärungsansätze und Implikationen für Politik und Institutionen, die Präventionsinterventionen gestalten.

## Einleitung

Menschen reagieren oft mit einem gewissen Maß an Widerstand auf Versuche, ihr Verhalten zu verändern [2]. Bis zu einem gewissen Punkt ist das wichtig und hilfreich, um sich vor Manipulations- und Beeinflussungsversuchen wie z. B. durch Werbung zu schützen. Der Impuls zum Widerstand kann aber dann dysfunktional oder sogar gefährlich werden, wenn er dazu führt, dass objektiv nötige und sinnvolle Schutzmaßnahmen unterlassen oder sogar aktiv bekämpft werden. Im Kontext der COVID-19-Pandemie kann und konnte ein relativ hohes Ausmaß an aktivem Widerstand oder passivem Desinteresse gegenüber Präventionsmaßnahmen beobachtet werden. So zeigt die Gutenberg COVID-19-Studie (*N* = 4040), dass die sog. AHA-Regeln (Abstand halten, Hygiene beachten und im Alltag Maske tragen) oft nicht eingehalten werden: Während 53,1 % der Befragten angaben, immer bzw. fast immer auf den Mindestabstand von 1,5 m zu haushaltsfremden Personen zu achten, teilten 44,8 % der Befragten mit, dass sie nur häufig darauf achten [32]. Eine andere Untersuchung ergab, dass im Juli 2021 noch 30 % der Befragten Verärgerung bzw. Reaktanz bezüglich der Schutzmaßnahmen empfanden und 12 % der Befragten sogar bereit waren, dagegen zu demonstrieren [30]. Die Zunahme polarisierender Positionen und aggressiver Diskurse bezüglich Coronaschutzmaßnahmen war auch in den sozialen Medien sichtbar [21]. So führte die im August 2021 gestartete TikTok-Kampagne der Bundesregierung z. B. zu derart viel Kritik und sogar Morddrohungen gegenüber einzelnen Influencer*innen, dass viele Spots nur nach wenigen Tagen zurückgezogen wurden [31].

Ziel dieses Beitrags ist es, Phänomene wie Abwehr, Resistenz oder Widerstand als antizipierbare Reaktionen auf Präventionsbotschaften näher zu beleuchten und den folgenden Fragen auf den Grund zu gehen: Wie sind solche Phänomene erklärbar? Welche präventiven Möglichkeiten der Minimierung gibt es? Welche Empfehlungen sind für eine abwehrsensible Gesundheits- und Risikokommunikation auszusprechen? Die Beantwortung dieser Fragen erfolgt auf der Basis einer Recherche des interdisziplinären Forschungsstands und einer Aufbereitung der wesentlichen Erkenntnisse für die praktische Gesundheits- bzw. Präventionskommunikation.

## Erklärung des Phänomens Abwehr

Die wissenschaftliche Beschäftigung mit dem Phänomen der Abwehr von Botschaften hat eine lange Tradition und erfolgte in verschiedenen Disziplinen. Besonders einflussreich waren die psychoanalytisch orientierten Arbeiten von Siegmund Freud, die von seiner Tochter Anna fortgeführt wurden [8]. In psychologischen und kommunikationswissenschaftlichen Forschungsarbeiten finden sich abwehrbezogene Phänomene teilweise anderen Bezeichnungen wie z. B. „Reaktanz“, „selektive Botschaftsvermeidung“ oder „Eskapismus“. Nachfolgend verstehen wir unter Abwehr oder Widerstand spontan und unbewusst ausgelöste Reaktionen auf gesundheitsbezogene Botschaften, die eine bewusste Auseinandersetzung mit einer als für das emotionale Wohlbefinden oder das Selbstwertgefühl zu bedrohlich erachteter Botschaft verhindern sollen. Das funktioniert überwiegend durch eine Ablenkung der Aufmerksamkeit von bedrohlichen Botschaften oder unseren emotionalen Reaktionen darauf oder durch eine verzerrte Realitätswahrnehmung [4]. Grundsätzlich kann sich Abwehr in vielen Formen äußern, so beispielsweise [11]:

1. Verleugnung oder Verdrängung von Risiken,
2. Rationalisierung des eigenen Risikoverhaltens, unrealistischer Optimismus,
3. aktive Vermeidung einstellungsdissonanter Botschaften,
4. aktive Suche nach einstellungskonformen Informationen und Personen,
5. einseitig-selektive Interpretation von Botschaften,
6. Angriffe auf Botschaftsinhalte, Gegenargumentieren,
7. Quellenabwertungen, Aggression gegenüber Andersdenkenden,
8. Ausführung des entgegengesetzten Verhaltens (Bumerangeffekt).

Die Befürwortung oder Ablehnung von Vorschriften oder Empfehlungen wie den AHA-Regeln hängt von vielen *individuellen, sozialen* und *kulturellen Faktoren* sowie *Charakteristika der jeweiligen Schutzmaßnahmen* ab. Diese Faktoren interagieren miteinander, aber sie lassen sich auch durch eine entsprechende Risiko- bzw. Gesundheitskommunikation in einem gewissen Ausmaß beeinflussen. Grundsätzlich ist immer davon auszugehen, dass Menschen eine mehr oder weniger stark ausgeprägte angeborene Tendenz haben, auf *subjektiv wahrgenommene Einschränkungen ihrer Wahlfreiheit oder Autonomie* mit Ablehnung zu reagieren [2]. Diese auch als „Reaktanz“ bekannte Reaktion tritt spontan und automatisch auf, wird also nicht bewusst-reflektiert initiiert. Das bedeutet nicht, dass Widerstand nicht auch das Ergebnis einer rationalen Abwägung der Vor- und Nachteile von Schutzmaßnahmen sein kann. Allerdings fokussiert dieser Beitrag bewusst auf Widerstand bzw. Abwehr im klassischen Verständnis von Reaktanz, also direkte, automatische Impulse zur Ablehnung. Dabei lässt sich Reaktanz auf psychologischer Ebene als eine Kombination von Verärgerung und Gegenargumentieren verstehen [22]. Reaktanz bewirkt, dass die kritisierten bzw. vermeintlich oder tatsächlich eingeschränkten Werte oder Verhaltensweisen (z. B. Feiern mit Freunden trotz Lockdown) als *attraktiver wahrgenommen *werden als vor der Kommunikation, in der diese Aspekte zur Sprache kamen. Sie kann im schlimmsten Fall dazu führen, dass Individuen ihre Freiheit bewahren bzw. Autonomie wiederherstellen wollen, indem sie das beanstandete Verhalten stärker ausführen als vor der Ansprache. Solche *Bumerangeffekte* sind als direkte Folge von Präventionskommunikation wissenschaftlich gut bestätigt [3, 10], aber ethisch höchst problematisch. Drei wesentliche Auslöser für Abwehrreaktionen sind:

1. wahrgenommene Einschränkung der persönlichen Freiheit,
2. wahrgenommene Bedrohung des emotionalen Wohlbefindens oder Selbstwertgefühls,
3. wahrgenommene Überforderung durch die Informationen.

Bei diesen Auslösern handelt es sich ausnahmslos um *subjektive Einschätzungen*, was impliziert, dass es ausschließlich auf die *individuellen *Einschätzungen der Botschaftsempfänger*innen ankommt. Ein gewisses Maß an Widerstand kann folglich bei allen Präventionsempfehlungen erwartet werden – und sollte entsprechend antizipiert und durch eine entsprechende Gestaltung der Kommunikation minimiert werden. Aus der Perspektive der Kommunikator*innen von Präventionsbotschaften sind ausgelöste Abwehrprozesse fast immer nachteilig. Denn es besteht eine gewisse Wahrscheinlichkeit, dass trotz gut gemeinter Intentionen und hohem Ressourceneinsatz das Gegenteil des eigentlichen Ziels erreicht wird. Der so ausgelöste Widerstand kann die Wahrscheinlichkeit reduzieren, dass sich die entsprechende Zielgruppe in Zukunft überhaupt oder unvoreingenommen mit vergleichbaren Botschaften auseinandersetzt und erhöht die Wahrscheinlichkeit von Aktivismus gegen die Schutzmaßnahmen [19]. Zur Ermittlung möglicher Einflussfaktoren wurde im Zeitraum von Juli bis September 2021 eine Literaturanalyse in PubMed durchgeführt. Als Suchbegriffe diente eine Kombination aus MeSH-Terms und Freitext. Verwendet wurden die Suchbegriffe: „covid-19“, „pandemic“, „reactance“, „psychological reactance“, „health behavior“, „rules“, „physical distancing“. „social distancing“, „decision making“. Ergänzt wurde die Literatursuche durch eine Recherche relevanter COVID-19-Studien, die im Zeitraum 2020 bis 2021 erschienen sind.

## Individuelle Einflussfaktoren

Die bereits geschilderten Befunde machen deutlich, dass Menschen in unterschiedlichem Maße „dispositionell“ reaktant sind, also situationsübergreifend unterschiedlich stark abwehrend auf wahrgenommene Einschränkungen reagieren. Studien belegen, dass Reaktanz als Persönlichkeitsmerkmal („trait“) auch die Bereitschaft zur Einhaltung von COVID-19-Empfehlungen beeinflusst (z. B. [6]). Dabei ist zu berücksichtigen, dass es innerhalb der menschlichen Entwicklung Phasen (wie z. B. die Pubertät) gibt, in der Reaktanz stärker ausgeprägt ist. Neben dieser dispositionellen Reaktanz können weitere Persönlichkeitsmerkmale mit einer Ablehnung von Schutzmaßnahmen verbunden sein, beispielsweise Einsamkeit [23], Glaube an Verschwörungstheorien, Misstrauen in offizielle Informationsquellen oder Affinität für komplementäre bzw. alternative medizinische Zugänge [27]. Informationen zu Gesundheitsbedrohungen wie COVID-19, die *negative Assoziationen* (z. B. zu schwerwiegenden Erkrankungen, dem eigenen Tod oder dem Verlust von Angehörigen) hervorrufen, können Auslöser für mögliche Abwehrreaktionen sein. Der Hinweis darauf, dass *Einschränkungen der Freiheit* oder *Verhaltensänderungen* im Sinne präventiver Maßnahmen nötig werden, verstärkt das Potenzial für die spontane Abwehr zusätzlich. Je nach Person kann es weitere bedrohliche Assoziationen geben, etwa die Angst vor dem Verlust des Arbeitsplatzes durch den Lockdown, die Angst vor sozialer Isolation oder die Angst vor Spritzen beim Impfen. Falschinformationen können weitere Trigger für Abwehr enthalten, indem sie sachlich nicht begründete Ängste vor Nebenwirkungen der Impfung oder vor einem totalitären Staat fördern. Entscheidend ist daher, dass offizielle Institutionen und Personen durch eine möglichst verständliche, professionelle und konsistente Kommunikation Irritationen und Verunsicherungen bestmöglich vermeiden, um das epistemische Vertrauen in diese Informationsquellen eher zu stärken als zu reduzieren.

Wie bereits ausgeführt, sind das Ausmaß subjektive wahrgenommener Einschränkungen bedeutsamer Freiheiten, das Maß der wahrgenommenen Bedrohung des Selbstwertgefühls bzw. des emotionalen Wohlbefindens sowie der antizipierten Überforderung durch die Informationen für das Entstehen bzw. Nicht-Entstehen von Reaktanz entscheidend. Der Coronavirus, die Pandemie, der Lockdown, die Intensivstationen usw. können sehr bedrohliche und potenziell überlastende Assoziationen hervorrufen. Vor diesem Hintergrund wird verständlich, wenn Menschen den Schutz ihres Selbstwertgefühls oder vor einer emotionalen Überforderung dem Schutz vor einem bedrohlichen Virus vorziehen. Aus der Forschung zum Umgang mit Stress ist bekannt, dass *kurzfristige *Vermeidungsstrategien hilfreich sein können, um zunächst die Emotionen (z. B. Angst) zu regulieren und sich zu beruhigen. Erst nach erfolgter Emotionsregulation kann eine fundierte Auseinandersetzung mit den konkreten Risiken und Bewältigungsmöglichkeiten erfolgen [13]. Langfristige Vermeidungsstrategien sind jedoch oft dysfunktional, da sie eine Auseinandersetzung mit dem Stressor verzögern und die eigentliche Ursache der Bedrohung bestehen bleibt. Selbst ohne Hinweis auf eine Bedrohung haben Präventionsbotschaften immer auch ein gewisses Potenzial zur Auslösung von Abwehr, da sie auf eine *Änderung des Verhaltens* abzielen. Menschen haben nicht nur ein starkes Bedürfnis nach *Autonomie* und *Kompetenzerleben* [5], weshalb Ratschläge von Dritten kritisch sind. Darüber hinaus haben Menschen ein Bedürfnis nach *Konsonanz*, d. h. sie sehen ihre Werte, Einstellungen, Wünsche und Verhalten gern in Übereinstimmung [7]. Kommunikative Versuche, Einstellungen und Verhaltensweisen zu ändern oder entsprechendes Wissen über Gesundheitsbedrohungen zu vermitteln, können folglich einen als unangenehm erlebten Spannungszustand bewirken. Dieser Spannungszustand wird umso stärker erlebt, je ausdrücklicher betont wird, dass eine Änderung des bestehenden Verhaltens notwendig ist. Die ausgelöste „kognitive Dissonanz“ zwischen Wissen, Einstellungen und Verhalten kann durch eine Vermeidung von Informationen über die Bedrohung durch COVID-19 abgebaut werden [26], die naturgemäß nicht im Interesse der Präventionskommunikation ist.

## Kulturelle und soziale Einflussfaktoren

Wie stark die beschriebenen individuellen Wahrnehmungen und Dispositionen Abwehr auslösen, hängt stark vom sozialen Kontext ab. Menschen vergleichen sich nicht nur mit anderen Menschen, sondern orientieren ihr Verhalten stark nach ihrem sozialen Umfeld. Entscheidend ist daher, welche Interpretationen und welches Verhalten Vorbilder, etwa Autoritären oder anderweitig anerkannte Personen (z. B. Influencer*innen) als *Rollenmodelle* zeigen. Gruppenprozesse spielen hierbei eine enorm wichtige Rolle: Selbst wenn nur eine Minderheit der deutschen Bevölkerung (14 %, *n* = 14.433) harte Distanzierungsregeln bei einer baldigen Lockerung ablehnten [9], kann eine „laute“ und gut vernetzte Minderheit die Wahrnehmung auslösen, dass ein substanzieller oder sogar überwiegender Teil anderer Personen ihre Meinung teilt und ggf. sogar eine „Schweigespirale“ bewirken, bei der Menschen ihre Auffassung seltener und vorsichtiger vertreten, da sie davon ausgehen, eine Minderheitsmeinung zu vertreten [29]. Sinnvoll ist es daher, genau zu beobachten, wie Minderheits- und Mehrheitspositionen in den Massenmedien und sozialen Medien kommuniziert und reproduziert werden. Das Ausmaß der Reaktanz hängt zudem davon ab, ob es sich eher um eine individualistische oder kollektivistische Gesellschaft handelt [16]. Kollektivisten fühlen sich aufgrund einer hohen Verbundenheit mit der Gruppe durch Einschränkungen weniger bedroht. Für Individualisten hingegen sind Freiheit und Unterscheidungskraft sehr wichtig, entsprechend fühlen sie sich von Einschränkungen auch deutlich eher bedroht [16].

## Einflussfaktoren präventiver Interventionen

Angesichts der beschriebenen Auslöser ist mit einer umso stärkeren Abwehr zu rechnen, je intensiver das *Ausmaß der wahrgenommenen Restriktionen* ist. So zeigte sich in einer szenariobasierten Studie, dass die Lockdown-Länge die Akzeptanz von Restriktionsmaßnahmen negativ beeinflusst [9]. Neben der Lockdown-Länge führten Gewöhnungseffekte sowie die Abnahme einer virusbedingten Sorge – ausgelöst durch eine reduzierte Medienberichterstattung bzgl. COVID-19-Risiken sowie zunehmende Berichterstattungen von Lockerungen – ab März und April 2020 dazu, dass die Akzeptanz von Präventionsmaßnahmen stetig abnahm [25]. Erneut zu betonen ist, dass es primär auf die *subjektive Wahrnehmung* der Maßnahmen ankommt, die stark durch die Art der Kommunikation beeinflusst werden kann. Das verdeutlicht die große Bedeutung einer präventionsförderlichen Kommunikationslandschaft, die bezüglich des *Agenda Settings* [18], des *Wordings* und *Framings* [17] der COVID-19-Risiken und Schutzmaßnahmen sensibel bezüglich unintendierter Effekte ist.

## Bausteine einer abwehrsensiblen Präventions- und Gesundheitskommunikation

Ob und in welchem Ausmaß Informationen Abwehr und Widerstand auslösen, hängt sowohl von den zu übermittelnden Informationen als auch der Art ihrer Kommunikation ab. Einige grundlegende kommunikative Anforderungen werden nachfolgend kurz zusammen:

### Professionelle Gestaltung

Vorausgesetzt wird zunächst, dass jede Kommunikation in formaler, inhaltlicher, visueller und sprachlicher Hinsicht hohen professionellen Standards genügt. Dies ist nötig, damit nicht bereits auf dieser frühen Ebene der Rezeption z. B. aufgrund von nicht erklärten Fachbegriffen, qualitativ schlechten Abbildungen oder Rechtschreibfehlern Zweifel an der Kompetenz der Kommunikator*innen entstehen, die eine Abwehr fördern.

### Respektvoll wertschätzende und stigmasensible Grundhaltung

Nicht nur aus Gründen der Höflichkeit, sondern auch aufgrund der Rolle des Selbstwertgefühls als Auslöser von Abwehr sollte Kommunikation stets so respektvoll und wertschätzend wie möglich angelegt sein. Häme und abwertende Bezeichnungen wie z. B. „Covidioten“ sind wenig geeignet, um skeptische Personen von der Sinnhaftigkeit von Schutzmaßnahmen zu überzeugen oder eine sachliche Informationsaufnahme zu fördern, sondern bewirken eher Ablehnung und Proteste. Aus demselben Grund sollte stigmasensibel kommuniziert werden, also versucht werden, bestehende Vorurteile oder Benachteiligungen keinesfalls – auch nicht unbeabsichtigt [24] – zu reproduzieren oder zu verstärken, sondern eher abzubauen.

### Vertrauenswürdige Quellen, Urheber*innen, Kommunikator*innen

Die Kommunikator*innen bzw. Quellen müssen in den jeweiligen Zielgruppen als vertrauenswürdig gelten, da die entsprechenden Informationen anderenfalls kaum beachtet bzw. vermieden oder nicht ernst genommen werden.

### Tailoring und Targeting

Menschen sind verschieden und es ist sinnvoll, das bei der kommunikativen Ansprache zu berücksichtigen. So sind Menschen nicht nur unterschiedlich dispositionell reaktant (vgl. Abschn. „Individuelle Einflussfaktoren“), sondern haben auch unterschiedliche Fehlwahrnehmungen über COVID-19, andere soziale Umfelder, abweichende Mediennutzungen und Unsicherheiten, verschiedene Barrieren für eine Verhaltensänderung usw. Daher ist anzuraten, die Kommunikation nach Möglichkeit für bestimmte Zielgruppen („targeting“) oder sogar Einzelpersonen („tailoring“) zuzuschneiden, statt einen „one-fits-all-approach“ zu verwenden.

### Evidenzbasierte Kommunikation von Risiken und Bewältigungsmöglichkeiten

Da Bedrohungen der Gesundheit bzw. des Lebens, des Selbstwertgefühls und des emotionalen Wohlbefindens psychisch belastend bzw. überlastend sein können und dadurch Abwehr auslösen, müssen bedrohliche Aspekte besonders vorsichtig adressiert werden. Bei der Kommunikation ist zu berücksichtigen, dass nicht nur (1.) das eigentliche Gesundheitsrisiko (z. B. eine COVID-19-Infektion), sondern auch (2. ) die Auseinandersetzung mit Informationen hierzu sowie (3. ) das Ausführen der empfohlenen Schutzmaßnahmen bedrohlich für das Selbstwertgefühl und emotionale Wohlbefinden sein können. Eine einseitige Betonung der körperlichen Risiken ist oft weniger erfolgversprechend als eine Kommunikation, die auch psychologische, soziale, emotionale, finanzielle sowie ggf. rechtliche Chancen und Risiken berücksichtigt und bei Bedarf betont.

Dem „extended parallel process model“ (EPPM; [33]) zufolge kommt es bei der Kommunikation über Risiken weniger auf den Aspekt der *Schwere der Konsequenzen* (z. B. Tod) an als darauf, dass die Kommunikation eine *persönliche Betroffenheit* bzw. Vulnerabilität deutlich macht. Das ist oftmals die eigentliche Herausforderung, da Menschen als Folge eines unrealistischen Optimismus oder von Abwehrprozessen wie Verdrängung oder Verleugnung ihre persönliche Betroffenheit nicht anerkennen. Zwei weitere subjektive Wahrnehmungen entscheiden dem EPPM zufolge darüber, ob Menschen im Falle einer wahrgenommenen Bedrohung präventiv oder abwehrend reagieren: die Einschätzungen, inwieweit die empfohlenen Schutzmaßnahmen tatsächlich *leicht umsetzbar* sind („Selbstwirksamkeit“) und inwieweit diese tatsächlich *wirksam zur Abwendung der Gesundheitsbedrohung* sind („Ergebniswirksamkeit“). Nur wenn die Selbstwirksamkeit und die Ergebniswirksamkeit (im Falle einer wahrgenommenen Bedrohung und persönlichen Betroffenheit) als sehr hoch bewertet werden, ist eine Schutzreaktion wahrscheinlich. Falls die Kommunikation hingegen Angst erzeugt, da primär die Existenz einer persönlichen Bedrohung wahrgenommen wird, aber keine leicht ausführbare und wirklich effektive Schutzmaßnahme, ist dem EPPM zufolge mit Abwehrprozessen zu rechnen. Diese empirisch gut bestätigte Annahme verdeutlicht, dass die *Einfachheit* der empfohlenen Schutzmaßnahme und deren *Wirksamkeit* so klar wie möglich kommuniziert werden müssen.

### Emotionale Appelle

Bezüglich der Verwendung emotionaler Appelle sollten die bisherigen Ausführungen verdeutlicht haben, warum reine Furchtappelle wenig Erfolg versprechend sind. Nicht nur wirken sie manipulativ und polarisierend, ihr negativ bedrohlicher Charakter fördert Abwehr und macht fraglich, dass ausreichend Selbst- und Ergebniswirksamkeit suggeriert werden kann. Auch Scham- oder Schuldappelle sowie injunktive moralische Appelle (Belehrungen bzgl. des „moralisch richtigen“ Verhaltens) sind eher abwehrförderlich. Positive Appelle sind vergleichsweise unproblematisch bezüglich Abwehr, da sie im Idealfall keine Bedrohung des Selbstwertgefühls bedeuten und gerne in sozialen Netzwerken geteilt (statt vermieden) werden, was insbesondere für Humorappelle gilt. Zudem können positive emotionale Reize als eine Art vorgeschalteter „Puffer“ wirken, um sich emotional für eine Auseinandersetzung mit Bedrohungen zu wappnen. Positiv können Appelle wirken, die zwar eine persönliche Betroffenheit vermitteln, allerdings durch eine positive und ermutigende Ansprache die Motivation und die Selbstwirksamkeit stärken. Das gilt auch für soziale Appelle, in denen die Bedeutung des Einzelnen nicht fordernd, sondern anerkennend in den Vordergrund gestellt wird, um persönliche und gesellschaftliche wahrgenommene Bedrohungen abzuwenden.

### Framing-Strategien

Analog zu den bereits geschilderten Aspekten sind *Gewinn-Frames* (Hinweise auf Vorteile durch die empfohlenen Verhaltensänderungen) mit hoher Wahrscheinlichkeit effektiver als *Verlust-Frames* (Hinweis auf Nachteile durch das bisherige Gesundheitsverhalten). Darüber hinaus empfiehlt es sich, die Passung der empfohlenen Verhaltensänderung zu bestehenden Einstellungen, Werten oder Verhaltenstendenzen zu betonen (Konsonanz-Framing), statt den Eindruck entstehen zu lassen, dass diesbezüglich fundamentale Änderungen anstehen. Es sollte sowohl der persönliche als auch der kollektive Nutzen angesprochen werden, um die kollektive Verbundenheit zu stärken.

### Narrative Persuasion

Strategisch konstruierte Geschichten können sehr aufmerksamkeitsstark und überzeugend sein, wenn sie es schaffen, durch ihren Spannungsbogen die Rezipierenden in ihren Bann zu ziehen. In diesem Zustand der kognitiv-emotionalen „Transportation“ sind die Rezipierenden weniger kritisch reflektiert gegenüber den in der Narration passierenden Geschehnissen und impliziten Schlussfolgerungen, wodurch auch die Abwehr reduziert ist [20].

## Zusammenfassung und Schlussfolgerung

Ablehnung von Schutzmaßnahmen sind nicht ungewöhnlich, haben aber ihren Preis. Ziel dieses Beitrags war es, zu verdeutlichen, woher diese Abwehrreaktionen kommen und wie vielfältig sie sein können – aber auch, wie wichtig sie sind, um Menschen vor Manipulationen und Überforderungen zu schützen. Im Anschluss wurde diskutiert, durch welche Kommunikation Abwehrprozesse unabsichtlich gefördert werden und welche Aspekte für eine abwehrminimierende Kommunikation entscheidend sind. Das Spektrum kommunikativer Möglichkeiten der Botschaftsgestaltungen sind selbstredend wesentlich umfangreicher, als wir in diesem Beitrag darstellen konnten (vgl. z. B. [1, 12]); wir haben uns daher ausschließlich auf solche Aspekte beschränkt, die bei der Auslösung oder Vermeidung von Abwehr eine wichtige Rolle spielen.

Ganz grundsätzlich wird Kommunikation nur erfolgreich sein, wenn die Zielgruppe bekannt ist. Ohne ein präzises Wissen darüber, aus welchen (auch ggf. unbewussten) Motiven eine Person Präventionsmaßnahmen ablehnt, sind Versuche einer Überzeugung, das Gesundheitsverhalten zu überdenken, wenig erfolgversprechend. Bedacht werden sollte auch, dass emotionale Ansprachen wesentlich überzeugender sein können als rein rationale. Für wirklich schwierige Situationen lohnt sich ein Blick auf die von Knowles und Linn [14] formulierten Alpha- und Omega-Strategien zur Resistenzminimierung. Diese wurden hier nicht ausgeführt, da ihnen ein stärkerer manipulativer Charakter zugeschrieben werden kann und das Grundproblem woanders liegt: Bislang wurden die Risiken durch COVID-19 sowie die Wirksamkeit der Schutzmaßnahmen nicht ansatzweise so kommuniziert, dass auf den ersten Blick die Schwere der Erkrankung, die Wahrscheinlichkeit einer Betroffenheit und den Unterschied, den die Einhaltung der AHA-Regelungen oder eine Impfung machen würden, für alle Menschen gleichermaßen deutlich werden konnte. Abstrakte und aus methodischen Gründen stark schwankende Zahlen wie Inzidenzwerte oder die Anzahl positiv getesteter Neuerkrankter (ohne Angabe der Dunkelziffer bzw. der Höhe der vermutlich tatsächlich erkrankten Personen) sind schwieriger greifbar und für viele Menschen ungeeignet, um Risiken und die Effektivität von Schutzoptionen adäquat abschätzen zu können. Anders sieht es z. B. bei grafisch gut aufgearbeiteten Informationen aus, z. B. wie viele angesteckte Personen man im Laufe eines Tages begegnen könnte, mit welcher Wahrscheinlichkeit man selbst innerhalb der nächsten 4 Wochen erkranken könnte und mit welcher Wahrscheinlichkeit ein schwerer Krankheitsverlauf bzw. „Long COVID“ zu erwarten wären. Auch eine vertrauenswürdige zentrale Informationsplattform mit klaren Informationen dazu, was eine COVID-19-Infektion auf individueller wie gesellschaftlicher Ebene gesundheitlich, sozial, finanziell usw. bedeuten kann, fehlt bislang. Hier dürfen klare und unmissverständliche Darstellungen über die Effektivität von Schutzmaßnahmen nicht fehlen [15]. Wenn beispielsweise die *Washington Post* kürzlich mutmaßt, dass die Verbreitung von Coronafalschinformationen primär daran liegt, dass viele Amerikaner*innen schlecht in Mathematik sind [28], muss die Frage gestellt werden, warum die relevanten Informationen bislang noch nicht so kommuniziert wurden, dass sie von allen Personen leicht verstanden werden, auch oder gerade von Menschen mit geringem statistischen Wissen. Eine derart prägnante Darstellung reduziert nicht nur Unsicherheiten und Missverständnisse, sondern auch die Anzahl widersprüchlicher Informationen in der Bevölkerung. Die stärkere partizipative Einbeziehung relevanter unterschiedlicher – auch reaktanter – (Risiko‑)Zielgruppen bei der Gestaltung von Gesundheitskommunikation hilft nicht nur, verständlichere Informationen zu erstellen, sondern auch dabei, mögliche Auslöser für Widerstände rechtzeitig zu erkennen und abzuschwächen. Viele Fragen bleiben offen: Inwiefern wurden Lai*innen, Expert*innen aber auch Medienvertreter*innen bei der Gestaltung von der Gesundheitskommunikation adäquat einbezogen? Inwieweit werden alle Teile der Bevölkerung so informiert, dass sie eine tatsächliche Chance haben, die Bedrohung und Effektivität der Schutzmaßnahmen adäquat einzuschätzen und auf dieser Basis eine fundierte Entscheidung über ihr Gesundheitsverhalten zu treffen? In welchem Maße waren höher gebildete Bevölkerungsschichten, die statistische Angaben entweder besser interpretieren oder einen besseren Zugang zu ergänzenden Informationen oder Interpretationen hatten, im (eventuell lebensrettenden) Vorteil? Es ist nur eine Frage der Zeit, bis sich dies konkret abschätzen lässt. Zudem einschränkend ohnehin anzumerken ist, dass eine aufklärend motivierende Kommunikation nur sinnvoll ist, wenn es auch leicht möglich ist, die empfohlenen Maßnahmen umzusetzen (Stichworte: Verfügbarkeit Desinfektionsmittel oder Impftermine). Die Coronapandemie zählt zu den größten individuellen und gesellschaftlichen Herausforderungen unserer Zeit. Sie hat aber auch vor Augen geführt, wie weit entfernt wir noch von einer evidenzbasierte Gesundheits‑, Risiko- und Präventionskommunikation sind, die angemessen abwehr- und stigmasensibel ausgestaltet ist. Auch in der Theoriebildung und interdisziplinären Erforschung der relevanten Kommunikations- und Informationsprozesse verdient dieser Aspekt eine wesentlich stärkere Beachtung.

## Fazit für die Praxis

- Reaktanz ist ein natürliches menschliches Phänomen, dass bei Präventionsinterventionen unter Berücksichtigung zielgruppenspezifischer Aspekte proaktiv berücksichtigt werden sollte.
- Akteure müssen sich darüber im Klaren darüber sein, dass Abwehrmechanismen durch die Kommunikation sowohl gefördert als auch reduziert werden kann. Sie sollten die entsprechenden Auslöser hierfür kennen und durch eine konsistente, verständliche und adressatengerechte Kommunikation dazu beitragen, das epistemische Vertrauen in offizielle Informationskanäle zu stärken.
- In der Summe sprechen alle Erkenntnisse dafür, Aktivitäten der Gesundheits- und Präventionskommunikation sowohl evidenzbasiert als auch abwehrsensibel zu planen und umzusetzen.
